# Improved Nitrogen Use Efficiency and Greenhouse Gas Emissions in Agricultural Soils as Producers of Biological Nitrification Inhibitors

**DOI:** 10.3389/fpls.2022.854195

**Published:** 2022-04-01

**Authors:** Shah Saud, Depeng Wang, Shah Fahad

**Affiliations:** ^1^College of Life Sciences, Linyi University, Linyi, China; ^2^Hainan Key Laboratory for Sustainable Utilization of Tropical Bioresource, College of Tropical Crops, Hainan University, Haikou, China

**Keywords:** biological nitrifying inhibitors, nitrification, nitrous oxide, nitrogen use efficiency, plants, root exudates, microbial

## Abstract

Based on an analysis of the current situation of nitrogen fertiliser application, it is suggested that improving the nitrogen utilisation efficiency of crops is an important means of promoting the sustainable development of agriculture and realises the zero increase in chemical fertiliser application. Nitrate loss and nitrous oxide (N_2_O) emissions caused by nitrification and denitrification are the main reasons for the low utilisation rate of nitrogen fertilisers. N_2_O is a greenhouse gas that has caused a sharp increase in global temperature. Biological nitrification inhibition refers to releasing natural compounds that inhibit nitrification from plant roots. The natural compounds released are called biological nitrification inhibitors (BNIs), which specifically inhibit the activity of microorganisms in soil nitrification. Biological nitrification inhibitors can significantly improve rice (*Oryza sativa*), corn (*Zea mays*) and other crops by 5–10%, which can increase the nitrogen utilisation rate of corn by 3.1%, and reduce greenhouse gas N_2_O emissions. Compared with plants that do not produce BNI, the amount of N_2_O released can be reduced by up to 90%. The BNI released by Brachialactone (*Brachiaria humidicola*) accounted for 60–90% of the total inhibition of nitrification. In summary, biological nitrification inhibitors that inhibit nitrification, improve nitrogen utilisation and crop yield, and reduce greenhouse gas emissions play an important role. This paper reviews the plants known to release BNIs, reviews the plants known to inhibit soil nitrification but with unknown BNIs and further discusses the important role of bio nitrification inhibition in agricultural systems.

## Introduction

To meet the world’s growing food demand, humans apply large amounts of nitrogen fertilisers to agricultural systems. While promoting food production, application also causes serious environmental pollution, such as water eutrophication, groundwater nitrate pollution, nitrous oxide (N_2_O) nitric oxide (NO) and other greenhouse gas emissions (Canfield et al., 2010). Before the industrialisation of agricultural production, the nitrogen budget was in a relatively balanced state for a long time, that is, nitrogen fixation and deamination accured at basically the same levels. However, since the 1930s, the Earth’s nitrogen cycle has been disrupted (Dansgaard et al., 1993), especially by the Green Revolution from 1960 to 2009, which used industrial compound fertilisers to cultivate rice and corn. Although nearly 1/4 of the world’s food was produced, this seriously damaged the ecological environment (Broadbent et al., 1977; Matson et al., 1999). The global annual consumption of nitrogen fertiliser is nearly 1.5 × 108 t, strong nitrification leads to the loss of nearly 70% of nitrogen fertiliser (Galloway et al., 2008) and the annual direct economic loss is approximately 81 million US dollars (the price of urea is 0.54–0.80 US dollars/kg; Subbarao et al., 2012). It is estimated that by 2050, global nitrogen fertiliser application will double to 3.0 × 108 t per year (Subbarao et al., 2012), and the annual loss of nitrogen caused by nitrate nitrogen lost from agricultural systems will reach 6.15 × 107 t (Schlesinger, 2009). This will increase the risk of nitrogen fertiliser loss in agricultural systems and aggravate environmental pollution problems.

Therefore, a several problems related to agricultural production need to be solved, the most important of which is increasing agricultural productivity. Chemical fertilizer application is one of the most important means of improving agricultural productivity, and improving the utilisation rate of nitrogen fertiliser is one of the key issues (Martinez et al., 2010). Nitrogen is essential for high crop yields (Rawluk et al., 2001). The utilisation rate of nitrogen fertiliser for food crops in our country is 28–41%. The loss mainly occurs in the volatilisation loss of ammonia, the leaching loss of nitrate nitrogen and gas loss (Yang et al., 2018; Anas et al., 2020). Seventy percent of nitrogen fertilisers in agricultural production are lost due to nitrification and denitrification.

Nitrification refers to the process by which ammonia produced by amino acids is converted into nitric acid under aerobic conditions by the action of nitrous acid and nitric acid bacteria. After fertilisation, a large amount of nitrate nitrogen will be leached due to precipitation and other phenomena, which will cause the loss of nitrogen fertiliser in the soil. In addition, a large amount of intermediate products NO and N_2_O will be produced during the conversion of NH_4_^+^ to NO_3_^−^. N_2_O is a controlled greenhouse gas (Gary and Lincoln, 2000; Daniel et al., 2002; Ito et al., 2018; Anas et al., 2020; Wang et al., 2021a,b) second only to CO_2_ and CH_4_. Its global warming potential is 298–310 times that of CO_2_, and it stays in the atmosphere for a long time (Liu et al., 2019). Therefore, certain methods need to be used to inhibit nitrification, thereby improving the utilisation rate and reducing the loss of nitrogen fertiliser.

In the mid-1950s, the concept of nitrification inhibition was proposed to inhibit the activity of ammonia-oxidising bacteria (AOB) and ammonia-oxidising archaea (AOA) and their related enzymes in nitrification, thereby inhibiting nitrification and the process of converting NH_4_^+^. It effectively slows down to NO_3_^−^, thereby slowing down the accumulation of NO_3_^−^, the release of N_2_O gas and the phenomenon of NO_3_^−^ leaching, so that the soil can keep the NH_4_^+^ content as high as possible. There are currently two main types of nitrification inhibitors (NIs), namely, industrial nitrification inhibitors and biological nitrification inhibitors (BNIs). Common industrial nitrification inhibitors are CP (2-chloro-6-(trichloromethyl) pyridine), DMPP (3,4-dimethylpyrazole phosphate), dicyanamide (DCD), etc. (Table 1), but their cost is higher and the duration of action in the soil is short. In addition to industrial nitrification inhibitors (Sun et al., 2016; Wang et al., 2021a,b), certain plants can inhibit nitrification by releasing natural compounds. Therefore, further study regarding how plants inhibit nitrification and to apply nitrification inhibitors excreted by plants to agricultural production is worthwhile (Rice et al., 1960) found in an abandoned field in Oklahoma that the rate of plant invasion matched the nitrogen and phosphorus needs of plants, suggesting a strong relationship between the nitrogen and phosphorus needs of the plants and the process of successful plant establishment. (Rice, 1964, 1965; Rice and Pancholy, 1974). The future biological nitrification research direction of inhibitors is proposed to provide a scientific reference for regulating the nitrogen cycle, improving the nitrogen utilisation rate and reducing greenhouse gas emissions. Several studies have found that plants can release substances that inhibit nitrogen fixation and nitrify bacterial activity. *Ambrosia artemisiifolia* and *A. psilostachya* inhibited the nitrification process, and *Bromus japonicus* also had the same effect (Meiklejohn, 1968). It is proposed that some tropical grasses may release certain compounds that inhibit nitrification, but they are not recognised due to a lack of direct evidence (Moore and Waid, 1971). It was found that root secretion extract can reduce the nitrification rate in clay. Among them, the inhibitory effect of ryegrass (*Lolium perenne*) secretion is the most significant, although the relevant mechanism of its inhibition has not been confirmed (Lata et al., 2000). Studies have found that plants of the genus Hyparrhenia with different ecological types have a certain ability to inhibit nitrification, which proved that plants could directly affect nitrification (Subbarao et al., 2006), the concept of nitrification inhibitors. The ability to release nitrification inhibitors from plant roots to inhibit soil nitrification is called biological nitrification inhibition, and the natural compounds released by plants are called biological nitrification inhibitors. In recent years, research on biological nitrification inhibitors has attracted increasing scientific attention (Wang et al., 2021a,b).

**Table 1:** The biological nitrification inhibitors (BNIs) released from intact roots of various plant species.

Serial no.	Plant species	Total BNI released from four plants (ATU Day)	Specific BNI (ATU g^−1^ root dry wt. Day)
*Pasture grasses*			
1.	*Brachiaria humidicola* (Rendle) Schweick.	51.1	13.4
2.	*B. decumbens* Stapf	37.3	18.3
3.	*Melinis minutiflora* Beauv.	21.4	3.8
4.	*Panicum maximum* Jacq.	12.5	3.3
5.	*Lolium perenne* L ssp. Multiflorum (Lam.) Husnot	13.5	2.6
6.	*Andropogon gayanus* Kunth	11.7	7.7
7.	*B. brizantha* (A. Rich.) Stapf	6.8	2.0
*Cereal crops*			
8.	*Sorghum bicolor* (L.) Moench cv. Hybrid Sorgo	26.1	5.2
9.	*Pennisetum glaucum* (L.) R. Br. cv. CIVT	7.0	1.8
10.	*Oryza sativa* L. cv. Sabana 6	0	0
	*Oryza sativa* L. cv. Toyo	0	0
11.	*Zea mays* L. cv. Peter no. 610	0	0
12.	*Hordeum vulgare* L. cv. Shunrai	0	0
13.	*Triticum aestivum* L. cv. Norin-61	0	0
*Legume crops*			
14.	*Arachis hypogaea* L. cv. TMV 2	9.4	2.5
15.	*Glycine max* L. Merr. cv. Orinoquia 3	0	0
	*Glycine max* L. Merr. cv. Natsuroyosooi	0	0
	*Glycine max* L. Merr. non nodulating type—EN 1282	0	0
16.	*Vigna unguiculata* L. Walpers ssp. unguiculata cv. Caupi	0	0
17.	*Phaseolus vulgaris* L. (accession G 21212)	0	0
	LSD (0.05)	7.1	2.8

‘0’ activity indicates that the inhibitory effect is possibly below the detection limit of the assay system used. *Source*: Subbarao et al. (2007b).

Biological nitrification inhibitors are a key element in improving nitrogen utilisation and improving fertiliser quality. For example, wider use of urea coated with urease and nitrification inhibitors, controlled release fertilisers, improved timing of nitrogen fertiliser. By inhibiting nitrification, BNI can effectively improve the utilisation rate of nitrogen in the soil, reduce nitrogen leaching and N_2_O gas emissions, and is of greatly affect the earth’s biochemical nitrogen cycle (Ito et al., 2018; Liu et al., 2019). This paper reviews plants that are known to release biological nitrification inhibitors and plants that are known to inhibit soil nitrification but does not show what compounds of biological nitrification inhibitors they release, or their effectiveness in improving crop productivity and improving nitrogen utilisation and reducing N_2_O gas release, nor does it discuss future prospects of biological nitrification inhibitors in agricultural systems, or provide a reference for BNI to further give full play to its role in production, with a view to providing a theoretical basis for the development of biological nitrification inhibitors and their wider global application.

## The Mechanism and Identification of Biological Nitrification Inhibitors

**Figure 1:**
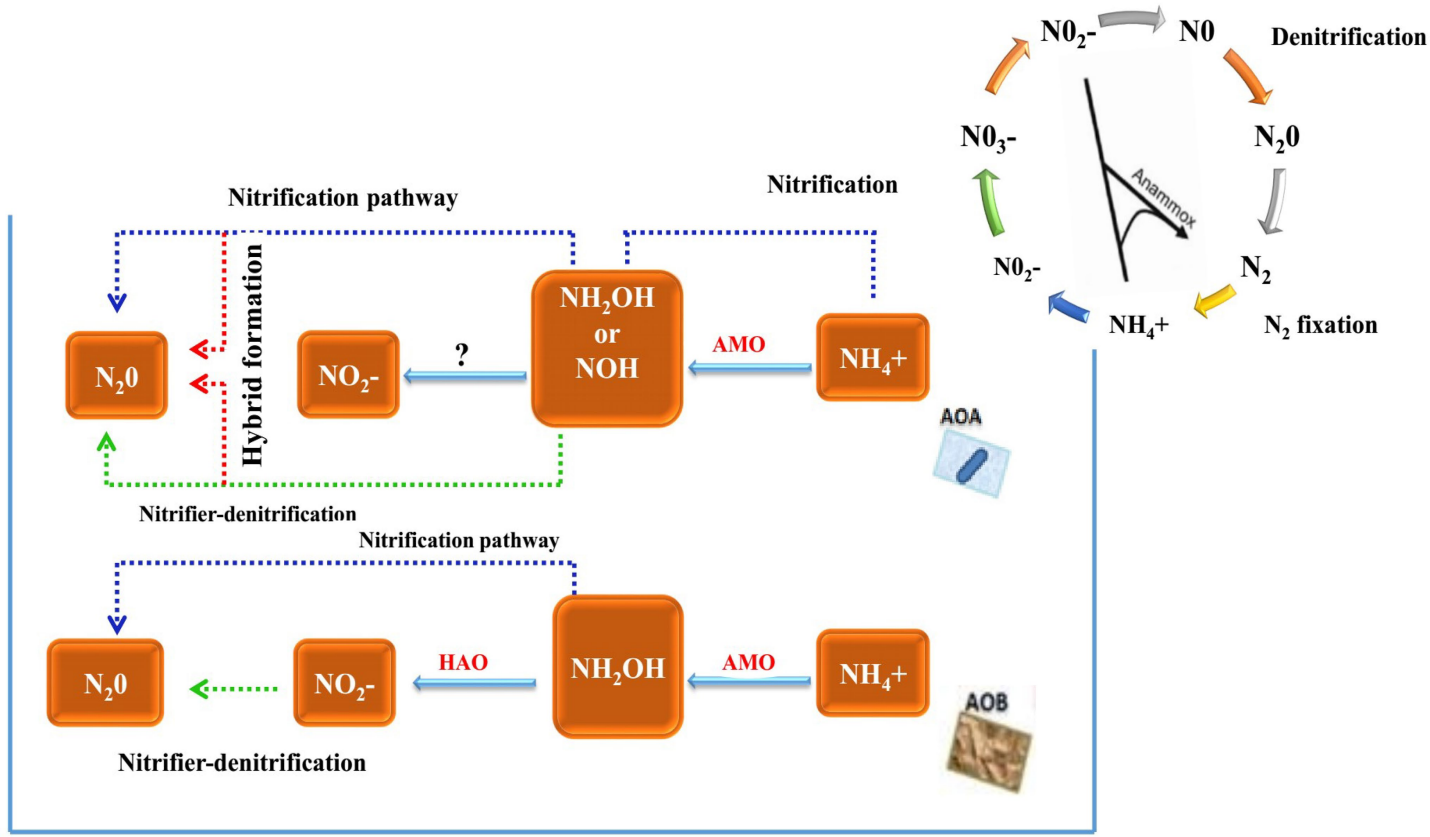
Historical timeline of discoveries on nitrification and ammonia-oxidising bacteria and major steps in the *N* cycle highlighting the AOB/AOA pathway within nitrification (Nsenga Kumwimba and Meng, 2019).

The inhibition of nitrification by BNI is mainly achieved by inhibiting the two pathways of ammonia mono oxygenase (AMO) and hydroxylamine oxidoreductase (HAO). The AMO pathway requires the participation of two nitrifying microorganisms, AOA and AOB, but the HAO pathway only requires one ammonia-oxidising microorganism, AOB. Whether its pathway has an impact on AOA remains to be confirmed. Therefore, BNI inhibits nitrification by inhibiting the AMO pathway (Figure 1).

The activity of AOA and AOB in the soil of their plant roots will be affected. For example, the soil of the roots of *Brachiaria humidicola* exhibits a lower abundance of AOB and AOA (Leininger et al., 2006; Leninger et al., 2016). BNI inhibits nitrification by inhibiting the HAO pathway. Only AOB activity is affected in the root soil of plants, such as *Leymus racemous* root soil, which exhibits a lower copy number of AOB genes (Subbarao et al., 2013). Currently, the main method that can detect and quantify the BNI activity of plant roots is the bioluminescence determination method of recombinant nitrosomonas bacteria (Subbarao et al., 2006). It can identify the characteristics of BNI traits in plants and ecosystems, and it has provided research on biological nitrification inhibitors with a major advance. The freeze-dried root secretions were added to 20 ml of 70% methanol solution and evaporated to dryness, and recombinant nitrosomonas and nitrous acid bacteria with fluorescent markers were inoculated. The nitrification inhibition ability was calculated by measuring the fluorescence quenching value of the root secretions (Subbarao et al., 2006). The process of the method is very fast, but since it cannot use commercially sold bacteria, recombinant bacteria must be fluorescently labelled under prescribed (PC2) laboratory conditions (O’Sullivan et al., 2016), proposed a simple and fast method for measuring BNIs in root secretions. This method can use *Nitrosospira europaea* and *N. multiformis* bacteria from the culture centre. All plants were grown using a hydroponic system, and the nutrient solution was changed every week. After 4 weeks of growth, root secretions were collected, rotated and evaporated to dryness, and the bacterial inoculation solution was added and incubated. Samples were collected every hour for a total of nine times. The collected samples were placed in a microplate, sulfonamide and nano were added by Griese reaction, vinylenediamine was chromatographed and then the concentration and nitrification rate of NO_2_^−^ were calculated by colorimetry at a wavelength of 490 nm. This method is an effective method for screening root secretions and can analyse the BNI traits of crops.

## BNI-Release Mechanisms in Plant Root Systems

Currently, plants releasing biological nitrification inhibitors include Brachium, *Sorghum bicolour*, *Oryza sativa*, *Pongamia* Glabra, and *Arbutus unedo*.

Brachium (arm) is the first tropical grass found to have strong adaptability to a low nitrogen environment, and it can inhibit nitrification. One of the bionitrifying inhibitors it releases is named ‘Brachialactone’, a cycloditerpenoid with a unique 5-8-5-metacyclic system and alactone ring.

It can be directly extracted from plant root secretions. The ability of this substance to inhibit nitrification accounts for 60–90% of the ability of arm-shaped grass to inhibit nitrification (Sahrawat, 1975; Subbarao et al., 2008; Sun et al., 2016). In addition, linoleic acid, α-linolenic acid and unsaturated fatty acids produced in the rhizosphere of African Wet Brachiaria and Brachiaria chinensis contain BNI activity. These three compounds can inhibit the activities of AMO and HAO at the same time, but the inhibitory effect on AMO higher and the duration of action can reach several months (Subbarao et al., 2009). Other plants with similar functions have similar inhibitory effects on AMO and HAO activities. Plants currently known to have the ability to release BNI are shown in Table 2. Byrnes et al. (2017) studied two African wet Brachiaria forages with different nitrification inhibition abilities and planted two African wet Brachiaria forages with different BNI secreting abilities for 29 days (the total *N* applied was 1.49 g/kg; soil the pH value is 6.2), the nitrification rate of the rhizosphere soil before and after planting was measured and the copy number of AOA and AOB was detected by quantitative PCR method; the results showed that the planting of Brachiaria humidicola cv. The N_2_O emission of the soil was 80 mg/m^2^, while the soil in which Brachiaria humidicola cv. Tully (BT) was planted with higher inhibition ability was only 32 mg/m^2^. The nitrification and denitrification in the BT rhizosphere soil the effect and abundance of AOA were significantly lower than those of BM, indicating that the forage with strong inhibitory ability played a leading role in inhibiting AOA activity (Byrnes et al., 2017). Genetic variation is a prerequisite for molecular breeding to change plant genotypes, and there is genetic variability in the ability of Brachiaria chinensis to release BNI.

**Table 2:** Bionitrification inhibitors isolated and identified from plants and their release amounts.

Plant	Isolated inhibitors of biological nitrification	BNI (ATU)
*Grasses*		
*Brachiaria humidicola*	Unsaturated fatty acid, linoleic acid, α-linolenic acid	51.1
*Brachiaria decumbens*	Linoleic acid, α-linolenic acid, unsaturated fatty acid	37.3
*Melinis minutiflora* Beauv.	Plant rhizosphere secretions	21.4
*Panicum maximum* Jacq.	Plant rhizosphere secretions	12.5
*Lolium perenne* Linn.	Plant rhizosphere secretions	13.5
*Andropogon gayanus* Kunth.	Plant rhizosphere secretions	11.77
*Brachiaria brizantha* Stapf.	Plant rhizosphere secretions	26.1
*Cereal*		
Sorghum bicolor *Oryza sativa* L. var. Sabana.	Sakuranetin, Sorgoleone 1,9-decanedio	8.7
*Legume crops*		
*Arachis hypogaea* L.	Plant rhizosphere secretions	–

Subbarao et al. (2007b) found that two nitrification inhibitors were released from the rhizosphere of sorghum: one inhibitor was a hydrophilic compound; the other was a hydrophobic compound, and the release rates of the two BNIs ranged from 10 to 25 ATU/(g d). In the pot experiment of sorghum planted for 30 days, soil BNI increased by 10 ATU/g, effectively reducing nitrification by 40%; the identification of hydrophilic BNI found that its main compound was sakuranetin, while the main component of hydrophobic BNI is a fatty acid compound with a benzene ring (sorgoleone), which can simultaneously inhibit the oxidation of hydroxylamine and ammonia oxidation (Subbarao et al., 2007b, 2013). The ability of sorghum to release biological nitrification inhibitors during the whole growth process increased continuously with the growth of sorghum, but the inhibitory effect of biological nitrification inhibitors continued to decrease with the growth of sorghum; the amount of hydrophilic and hydrophobic biological nitrification inhibitors secreted by sorghum. There was no significant difference, the only difference was that the amount of hydrophilic inhibitor released by the roots decreased significantly after 130 days of sorghum growth. Zakir et al. (2008) and Nardi et al. (2013) studied a kind of BNI secreted by sorghum, parahydroxyphenylpropionic acid (MHPP), and found that the secretion rate of MHPP increased with the increase of ammonium ion concentration. After treatment of sorghum roots with fusicoccin and the inhibitor vanadate, it was found that clostridia could increase the secretion of MHPP, while vanadate reduced its secretion in Table 2.

The soil at the roots of Brachycodon exhibits a lower abundance of AOB and AOA (Moreta et al., 2014). The BNI released by arm-shaped grass has a stronger inhibitory effect than the industrial nitrification inhibitor CP. Studies have found that in pastures where arm-shaped grass is grown for a long time, the biological nitrification inhibitors accumulated in its soil can be used in other crops grown here and can improve the soil NUE, which is of great significance in agricultural production (Nelson and Huber, 1992; Karwat et al., 2017).

**Figure 2:**
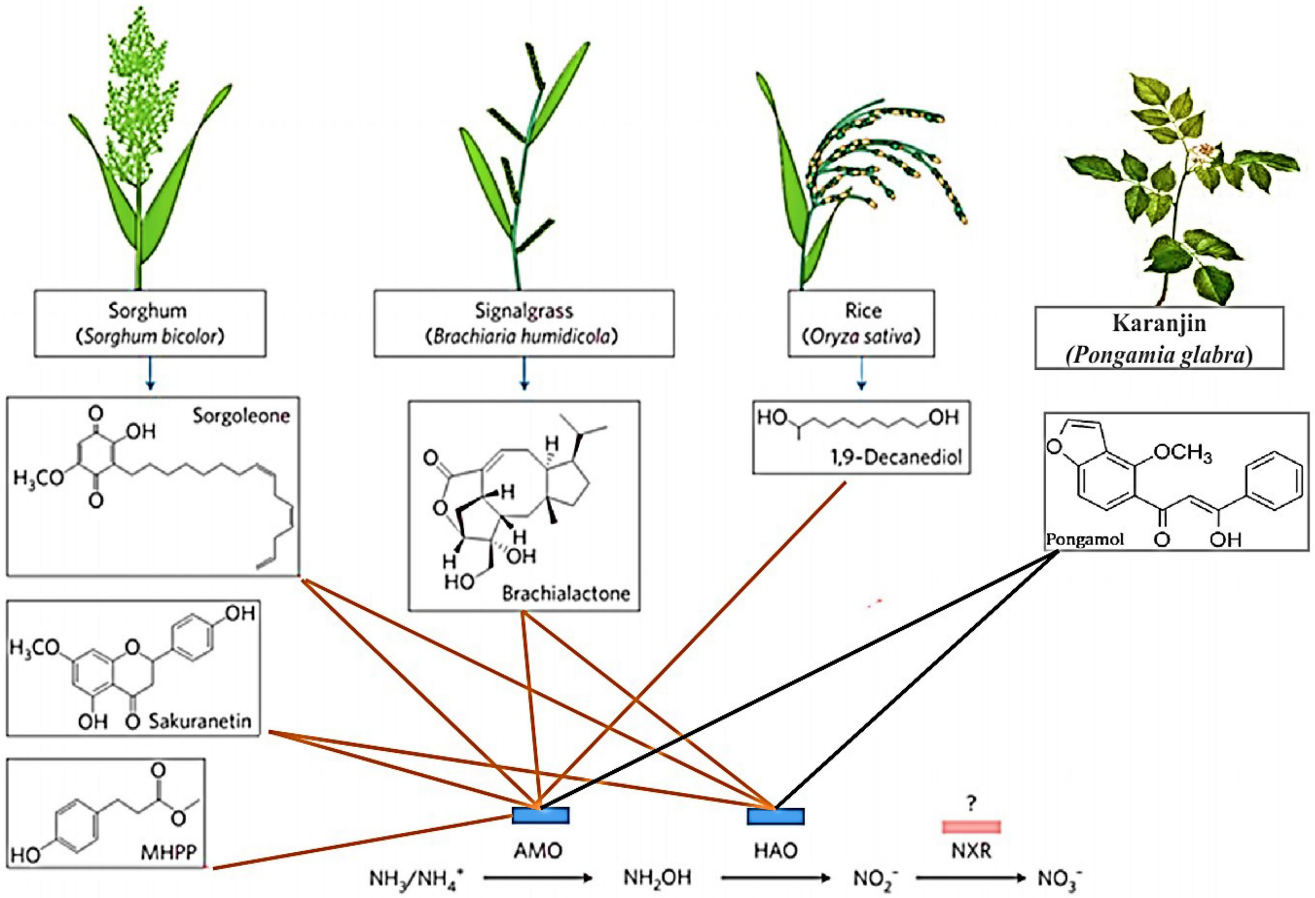
Biological nitrification inhibitors (BNIs) from root exudates and their enzyme targets. BNIs isolated from exudates isolated to date are shown, together with their source plants and their enzyme targets for catalysing nitrification. The red line represents known motion patterns, and the black line represents unknown motion patterns (Adopted from Coskun et al., 2017).

In cereal species, sorghum showed significant inhibition of nitrification. Its roots mainly release two biological nitrification inhibitors, hydrophilic BNIs and hydrophobic BNIs (Di et al., 2018). Sorghum root hydrophilic BNIs release mainly sorghum quinone, p-hydroxyphenylpropionate methyl ester (MHPP), whose scientific name is 4-hydroxybenzene-3-methylpropionic acid, which is a BNI with stronger inhibitory ability compared to other crops (Subbarao et al., 2007b, 2008). Both sorghum quinone and MHPP were extracted directly from root secretions, and MHPP was the first direct extraction of BNI from root secretions. In the past, BNI was extracted from plant tissue and soil (Coskun et al., 2017). When the nitrogen sources are ammonium nitrogen and nitrate nitrogen, both promote the secretion of nitrification inhibitors, but ammonium nitrogen has a greater impact on it. This may be because the synthesis of MHPP requires the precursor substance phenylalanine, and the synthesis of phenylalanine requires L-phenylalanine aminolyase. Ammonium ions regulate the secretion of L-phenylalanine aminolyase, so ammonium nitrogen strongly influences the secretion of MHPP (Zerulla et al., 2001; Tesfamariam et al., 2014). pH has a certain effect on the secretion of hydrophilic BNIs. When pH ≥ 5, the release rate of hydrophilic BNIs decreases. When pH ≥ 7, 80% of the secretion of hydrophilic BNIs is suppressed, but pH does not effect on the secretion of hydrophobic BNIs (Figure 2; Di et al., 2018; Wang et al., 2021b). In addition, the age of plant can also affect the secretion of BNIs. Therefore, by improving the soil into a habitat that is more suitable habitat for plant to secrete BNIs, plants can release more BNIs.

Rice is another cereal crop that been shown to possess the ability to significantly inhibit nitrification (Sun et al., 2016). Using the *in-situ* collection system of root secretions and GC-MS coupled with identification technology, a new type of biological nitrification inhibitor, 1,9-decanediol was identified from rice for the first time. It mainly inhibits nitrification by inhibiting the AMO stage, and it is clear that 1,9-decanediol is a natural substance secreted by the rice root system, especially it has a significant inhibitory effect on the nitrification of tidal ash soil. The inhibitory effect is greater than the industrial nitrification inhibition of DCD that is commonly used in agricultural and animal husbandry production. Rice secretions can reduce the rate of nitrification rate, but the inhibition rate of different rice varieties of rice. The exudation of nitrification inhibitors released by rice is positively correlated with rice’s absorption and preference for ammonium nitrogen, which provides an advantage for the utilisation nitrogen in rice. This can be used to develop and improve varieties in breeding (Sun et al., 2016).

BNI’s have not been found in Triticum aestivum, but it has been found in some wild-type wheat to have 80% of the ability to inhibit nitrification (Kishii et al., 2008). The root exudates of *Lolium perenne* Linn inhibited the formation of nitrate nitrogen and approximately 13.5 BNI (ATU) of the nitrogen in the soil was in the form of ammonia nitrogen. Significant inhibition of nitrification was detected in the soil of *Lolium perenne* Linn, where ammonium nitrogen was the source of nitrogen. Still no nitrate inhibition was detected in soil with a nitrate-nitrogen source (Subbarao et al., 2007a,c; Tanaka et al., 2010).

Plants that release biological inhibitors exist not only in grasses but also in woody plants such as Kalanja, Melia azedaeach and poplar. The neem tree is a tree species that has many benefits for humans. It can be used as medicine, to reduce erosion, and to reduce the greenhouse effect (Vietmeyer, 1992). Suggested that methanol extract from Chinaberry trees can inhibit nitrification (Sivasakthy and Gnanavelrajah, 2010) and can be obtained from the seeds of nitrifying bacteria, which has a significant inhibitory effect on the activity of nitrifying bacteria in the soil. In addition, the Karanja tree can release the following BNIs hydroxano-2, 3, 7, 8-flavone or 3-methoxy-2-phenyl [2, h]chromen-4-one (Sahrawat, 1975). The study found that after fertilising the Kalanja tree, its inhibitory effect will not increase in proportion to the applied dose. Still it will strengthen the presence of ammonium nitrogen and will not inhibit the conversion of nitrite to nitrate, but it inhibits the activity of ammonia-oxidising bacteria. Subsequently, it was found that there is a crystalline component in the seeds of the Kalanja tree that inhibits nitrification. There is an important structure in the molecule-furan ring (C_4_H_4_O), which mainly act by changing the activity of biological nitrification inhibitors (Sahrawat and Mukerjee, 1977; Majumdar, 2002). In the 30-day trial, it was found that the Kalanja tree inhibits nitrification by 65–75% and reduces N_2_O gas emissions by 92–95%, which can greatly improve the utilisation rate of nitrogen fertiliser. Bayberry produces catechins (C_15_H_14_O_6_·H_2_O) catechols (C_6_H_6_O_2_) biological nitrification inhibitor active substances during leaf litter decomposition, and they have a certain inhibitory effect on nitrification. The content of catechins and catechols in the soil of the roots of bayberry of the same volume is less than that released in leaf litter constitutes, so the main action of inhibition (Sahrawat, 1981; Castaldi et al., 2009).

Therefore, biological nitrification inhibitors are of indispensable significance to the global nitrogen cycle. From perspective of improving nitrogen utilisation and reducing environmental pollution, they play a great role. However, there are very few plants that possess discovered and extracted BNIs. More ways discovering and applying new BNIs need to be explored.

### Biological Nitrification Inhibitors Potential in Plants

Current studies have found that some plants can inhibit nitrification, but no active compounds have been extracted from them (Bowatte et al., 2016). The nitrification potential of 126 forage species was analysed as the basis for discovering BNIs to explore whether it can reduce N_2_O. It was found that Italian ryegrass (Lolium multiflorum) had the highest nitrification potential, while perennial ryegrass (*L. perenne*) had the lowest. Eleven kinds of grasses, three kinds of non-grasses and two kinds of legume crops were further studied. Italian ryegrass had the lowest release of N_2_O gas, and mountain sparrow wheat (*Bromus stamineus*) had the highest release, but there was no significant difference (Subbarao et al., 2007a; Bowatte et al., 2018). Both indicate that different plants have different soil nitrification capabilities. It is likely that certain substances released from plant roots affect the activity of soil microorganisms and thus affect nitrification (O’Sullivan et al., 2017). The effects of several common Australian weeds, such as wild radish (*Raphanus raphanistrum*), bromegrass (*B. diandrus*), wild oats (*Avena fatua*) and perennial ryegrass on nitrification under hydroponic conditions were studied. The results showed that radish root exudates inhibited nitrification most significantly, and its nitrification activity intensity was higher than that of Brachiaria wetland. Other plants also had a certain inhibitory ability (Wozniak et al., 1999; Mao et al., 2006; Chen et al., 2008). After the crude extract of Astragalus astragalus was applied to the soil at different rates and cultured for 30 days, it was found that the soil urease activity and denitrifase activity were significantly increased, and the soil nitrification rate was significantly reduced compared to the control group.

Although many plants have certain properties that inhibit nitrification, how to use this property in actual production needs to be further studied (Subbarao et al., 2007b, 2013). A gene that can inhibit nitrification was found in a close relative of wheat, and this related gene was introduced into wheat, resulting in many effective inhibitor compounds. In agricultural systems, the genetic improvement method of hybridisation can give wheat a certain ability to inhibit biological nitrification (Subbarao et al., 2007c). However, this method is only used in the same plant species, and no effects have been reported between different species.

### Significance of Biological Nitrification Inhibitors to Plants

**Figure 3:**
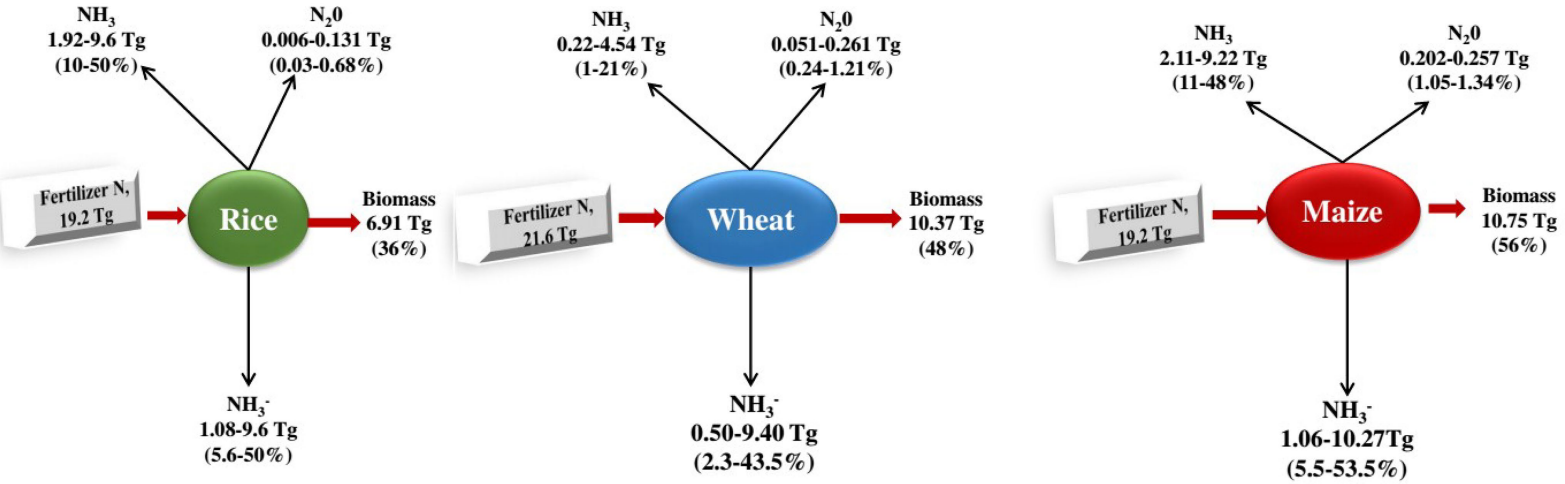
Nitrogen budgets of the three large plants. From the c. 120 Tg *N* yr1 fixed according to the Haber-Bosch process 2, 50% is applied to the three most important types of grain in the world, rice (16%), wheat (18%), and maize (16%; Ladha et al., 2016), which together are more than 60% of human caloric intake 120 and cover approximately 546 million hectares (36%) of the world’s arable land 33. It shows the global averages of fertiliser *N* recovery (the fertiliser *N* portion retained as biomass) for the three grain types 119. The remaining nitrogen is lost to the environment through NH_3_ volatilisation, NO_3_ leaching and runoff, denitrification (production of NO, N_2_O and N_2_ gases) and is also immobilised by other organisms or soils 30, 33, 121130. The percentage of nitrogen lost varies depending on the type of fertiliser and environmental factors, including temperature, wind speed, rain, and soil properties such as cation exchange capacity and pH (Coskun et al., 2017).

Biological nitrification inhibitors can reduce N_2_O emissions, slow nitrogen leaching, improve nitrogen utilisation efficiency and increase crop production. For example, Zhang et al. (2011) found that the total vegetable production treated with BNI was 163.2 t·(hm^2^ a)^−1^, which was 10.3 t·(hm^2^ a)^−1^ higher than that of the control (application of urea), and the application of BNI significantly increased agricultural production (Datta and Adhya, 2014; Figure 3).

It was found that the application of BNI ‘Nimin’ and Xanthoderma lucidum can significantly increase rice yield while reducing CH_4_ and N_2_O emissions per unit weight of rice (Sun et al., 2016; Cui et al., 2021) conducted experiments between sorghum and corn, respectively, and found that the expression of AOA and AOB were both sorghum < sorghum and corn intercropping < corn, and the corn yield increased by 5.6%, and its nitrogen fertiliser utilisation rate increased by 3.1%. Planting plants that release BNI and apply BNI extract can increase crop yield and nitrogen fertiliser utilisation, reduce greenhouse gas emissions (Zhang et al., 2015).

The *N* content of DCD, CP, and BNI combined with urea was compared to N_2_O emissions and nitrogen use efficiency (NUE). Compared to urea treatment, DCD treatment had no significant effect on N_2_O or agricultural NUE, while CP and BNI significantly reduced annual N_2_O emissions by16.5 and 18.1%, respectively; NUE increased by 12.6 and 6.7%, respectively, showing that BNI improves yield in vegetable ecosystem and reduces N_2_O emissions thus playing an important role (Luo et al., 2008; Rossiter-Rachor et al., 2009). N_2_O gas was measured after animal urine was applied to pastures in New Zealand. The results showed that the application of bovine urine would stimulate the emission of N_2_O gas. Therefore, the nitrification rate can be reduced by selecting grasses that can release BNIs in pastures. (Suter et al., 2006; Abalos et al., 2014). Whether different plant compositions would affect N_2_O emissions after applying animal urine was also studied. The results showed that plant richness does not reduce N_2_O emissions, and less gas is emitted in mixed plots with high fescue, so a targeted selection of plant species for land management can reduce N_2_O gas emissions. Whether in crop planting fields or grazing pastures, choosing plants that can release biological nitrification inhibitors can effectively improve nitrogen utilisation efficiency, reduce N_2_O gas emissions and increase crop yield.

It has not been established whether the release of biological nitrification inhibitors is due to their specific release or adaptation to the environment (Lata et al., 2004; Coskun et al., 2017). It is proposed that biological nitrification inhibition may be part of the adaptation mechanism for plants to preserve and use nitrogen effectively, and nitrogen deficiency may be an incentive to promote the evolution of biological nitrification inhibitors. Many plants can only observe the release of BNIs when the nitrogen source is NH_4_^+^ so plant root release inhibitors are a local phenomenon, limited to the part of the root in the NH_4_^+^ environment. They have not extended to the rest of the root system. The soil NH_4_^+^ mineralisation of soil organic nitrogen and nitrogen fertilisers can increase the activity of nitrifying bacteria (Robinson, 1963; Trenkel, 2007). The regulatory role of NH_4_^+^ in inhibitor synthesis and release suggests that this may be another trigger for the evolution of biological nitrification inhibitors (Subbarao et al., 2007b). In addition, although nitrification will produce some unfavourable phenomena, it is also necessary to consider that a certain degree of nitrification also has beneficial effects. For example, in rice cultivation, the coexistence of NH_4_^+^ and NO_3_^−^ plays a synergistic role in promoting rice growth (Kronzucker and Siddiqi, 1999; Kirk and Kronzucker, 2005).

## Summary and Future Perspectives

Research on BNIs is currently still in the early stages. For the past decade, BNIs have been extracted directly from the secretions of the root system of Brachiosia, sorghum and rice, suggesting that great strides have been made in this area. The presence of BNIs can improve nitrogen use efficiency, significantly improve grain productivity and reduce greenhouse gas emissions. In the current research, apart from further research on BNIs for food crops such as rice and sorghum, there has been little research on regarding plants. Therefore, there is still much room for exploring whether more plants can secrete BNIs. For example, BNIs have not been found in corn, an important food crop. However, it cannot be ruled out that all its varieties contain BNIs. Only with more research on related basic work can the use of BNIs be tapped to deal with the environmental degradation and agricultural problems facing the world today.

To solve this problem, further research is needed to investigate whether stimulation of breeding and growth conditions can alter the synthesis and release of BNIs, and how the precise release and optimization of BNIs can be controlled to control nitrification effectively (Subbarao et al., 2013). The BNI related genes from wheat relatives have only been successfully introduced into wheat, but how to transfer genes between different species remains to be further investigated. Few methods currently exist to detect and quantify the activity of BNIs. Due to the complex nature of the microorganisms in actual soil (Subbarao et al., 2013), these methods are limited to a single ammonia-oxidising bacterium, so scientists should focus on finding a better way to detect the activity of BNIs so that more BNIs can be discovered.

Better plants and varieties should be selected to provide technical support for agricultural production. The discovery of more bNIST-releasing forage and the introduction of this forage to pastures that produce high yields with low nitrification rates and reduce greenhouse gas emissions could also support pasture systems. Wang et al. (2021a,b) also proposed reducing the loss of nitrogen fertiliser in the soil through crop rotation or mixed crops to free BNI crops and encourage agricultural system development. In addition, one of the important research directions is to improve and cultivate more biological nitrification inhibitors with higher nitrification inhibition capacity through modern genetic and molecular techniques. It is also an important direction for future research to improve or update existing synthetic chemical nitrification inhibitors by using discovered BNIs.

With continuous improvement in the awareness of nitrogen utilisation, and with the encouragement and supervision of government agencies and relevant agricultural sectors in the future, which might be, similar to measures such as extensive subsidies and a global trading system that restricts carbon emissions and encourages low-carbon agriculture, the majority of farmers may use crop varieties with high nitrogen utilisation and low nitrogen loss or grow plants with high BNI in combination with general crops in pastures, thereby increasing nitrogen utilisation (Oita et al., 2016).

## Author Contributions

SS, DW, and SF: conceptualization. SS and SF: writing—original draft. DW and SF: writing—review and editing. All authors contributed to the article and approved the submitted version.

## Funding

This research was supported by the National Natural Science Foundation of China (project no. 32001468).

## Conflict of Interest

The authors declare that the research was conducted in the absence of any commercial or financial relationships that could be construed as a potential conflict of interest.

## Publisher’s Note

All claims expressed in this article are solely those of the authors and do not necessarily represent those of their affiliated organizations, or those of the publisher, the editors and the reviewers. Any product that may be evaluated in this article, or claim that may be made by its manufacturer, is not guaranteed or endorsed by the publisher.
